# Optimizing the pipeline of multipurpose prevention technologies: opportunities across women's reproductive lifespans

**DOI:** 10.3389/frph.2023.1169110

**Published:** 2023-05-30

**Authors:** Anna Bershteyn, Danielle Resar, Hae-Young Kim, Ingrida Platais, Saiqa Mullick

**Affiliations:** ^1^ Department of Population Health, NYU Grossman School of Medicine, New York, NY, United States; ^2^ Clinton Health Access Initiative, Boston, MA, United States; ^3^ Wits RHI, University of the Witwatersrand, Johannesburg, South Africa

**Keywords:** HIV, PrEP (pre-exposure prophylaxis), pregnancy, prevention, contraception

## Abstract

HIV/AIDS and maternal mortality are the two leading causes of death among women of reproductive age in sub-Saharan Africa. A growing body of research investigates opportunities for multipurpose prevention technologies (MPTs) that prevent unintended pregnancy, HIV, and/or other sexually transmitted infections (STIs) with a single product. More than two dozen MPTs are currently in development, most of them combining contraception with HIV pre-exposure prophylaxis, with or without protection from other STIs. If successful, such MPTs could offer women benefits at multiple levels: greater motivation for effective use; lower product administration burden; accelerated integration of HIV, STI, and reproductive health services; and opportunities to circumvent stigma by using contraception as a “fig leaf” for HIV and/or STI prevention. However, even if women find respite from product burden, lack of motivation, and/or stigma in contraceptive-containing MPTs, their use of MPTs will be interrupted, often multiple times, over the reproductive lifecourse due to desire for pregnancy, pregnancy and breastfeeding, menopause, and changes in risk. Interruptions to the benefits of MPTs could be avoided by combining HIV/STI prevention with other life-stage-appropriate reproductive health products. New product concepts could include combining prenatal supplements with HIV and STI prevention, emergency contraception with HIV post-exposure prophylaxis, or hormone replacement therapies for menopause with HIV and STI prevention. Research is needed to optimize the MPT pipeline based on the populations underserved by available options and the capacity of resource-constrained health systems to deliver novel preventative healthcare products.

## Introduction

HIV/AIDS and maternal mortality are the two leading causes of death among women of reproductive age in sub-Saharan Africa and in the lowest socioeconomic quintile globally (1). These sexual and reproductive health (SRH) burdens frequently overlap because HIV infections among women primarily occur in the context of unprotected sex with men.

Multipurpose prevention technologies (MPTs) are products that serve multiple SRH preventative care needs with one product, such as preventing unintended pregnancy, HIV, and/or other sexually transmitted infections (STIs) (2). As a single product, MPTs may reduce the number of product administration events required to meet SRH needs, e.g., as self-administered pills, vaginal inserts, or injections; or provider-administered injections, devices, or implants. For oral pills, evidence from HIV and other disease areas suggests that decreasing pill burden through “one pill, once a day” dosing is associated with substantially improved adherence (3–5). For injections, evidence from several injectable regimens (6), such as HIV PrEP (7), HIV treatment (8), and diabetes treatment (9), suggests greater user and provider satisfaction with regimens requiring fewer injections. Product satisfaction has been important determinant of adherence among users (10) and prescribing among providers (11).

Currently, the only available MPTs are condoms, which are non-discreet, difficult for women to negotiate, and less effective with typical use compared to available single-indication products (12–16). Van der Straten et al. randomized young women in South Africa and Kenya to try a placebo form of a pill, injection, or ring MPT for 1 month, then select a form to continue for another 2 months, and found that 85% of women reported preferring their MPT over condoms (17). Fortunately, the landscape of MPTs is poised for transformation. As of February 2023, there are 28 new MPTs in development, including pills, injections, implants, as well as several non-systemic product forms such as vaginal rings, films, and gels (18). A majority of these MPTs (18 of the 28) prevent pregnancy together with HIV and/or other sexually transmitted infections (STIs), while a smaller proportion combine HIV and non-HIV STI prevention.

**Figure 1:**
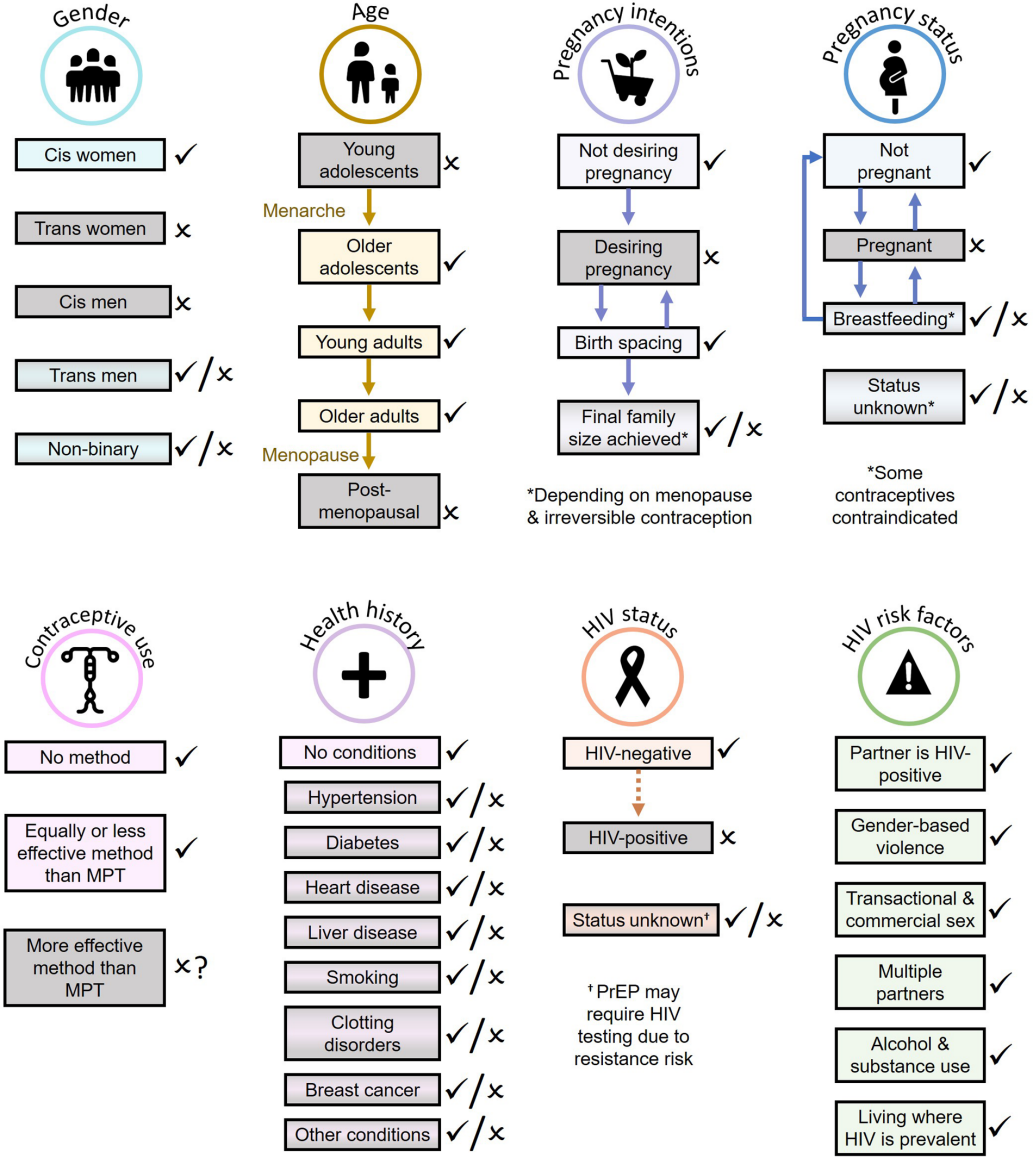
Populations most likely (light rectangles with check marks) and least likely (gray rectangles with x marks) to benefit from MPTs currently in development.

While many could benefit from MPTs under development, it is important to recognize that not all individuals in need of a combination product will be willing or able to benefit from MPTs (Figure 1). Some will be excluded from benefitting from the current product pipeline, while others will experience interruptions in MPT eligibility over their reproductive lifespan, e.g., when desiring pregnancy or pregnant yet still requiring HIV and/or STI prevention. Additionally, compared to single-indication products, more individuals are likely to be excluded from using MPTs due to the collective contraindications, side effects, and screening requirements of multiple combined products.

Exclusion of important and often vulnerable populations (e.g., pregnant women) from the benefits of MPTs have implications in both the ability to implement MPT delivery effectively, and implications for health equity. Multiple innovation frameworks recommend an equity lens incorporating both patient and provider perspectives on healthcare products and implementation methods. The Health Equity Implementation Framework combines implementation and healthcare disparities research methods to integrate characteristics of the innovation (e.g., a new MPT), patient factors, provider factors, and their health system, sociopolitical, societal, and economic contexts to guide innovations that improve both implementation and health equity (19). An innovation outcomes addendum to the widely-used Consolidated Framework for Implementation Research (CFIR) similarly integrates indicators from innovation recipients (patients), innovation deliverers (providers), and key decision-makers around the goal of equitable population impact (20).

Using these frameworks as a guide, this article reviews the potential benefits, gaps, and opportunities for MPTs across the lifespan, including: (1) women not wanting to get pregnant, (2) women actively trying to get pregnant, (3) pregnant and breastfeeding women, and (4) women approaching and experiencing menopause.

## Not currently desiring pregnancy

Women undergo multiple stages of need for pregnancy prevention—including young women not yet ready to begin a family, women wishing to space pregnancies, and women who have achieved their desired family size and do not desire additional pregnancies. Of these groups, adolescent girls and young women, who frequently do not yet wish to begin a family, bear a disproportionate burden of new HIV infections in sub-Saharan Africa and face unique challenges in preventing pregnancy and HIV.

Despite their elevated risk, young women tend to express greater concern about avoiding pregnancy than HIV, especially in the context of successful HIV treatment programs (21, 22). In trials of user-dependent HIV prevention products—pills, gels, and vaginal rings—younger women tend to exhibit lower product adherence (23–26). MPTs combining contraception and HIV prevention may unlock stronger motivation to use HIV prevention effectively (27).

For women who experience unanticipated events such as sexual assault, or whose prevention needs are anticipated but intermittent, future MPT product concepts might include combinations of emergency contraception plus post-exposure prophylaxis (PEP), or on-demand contraception plus risk-informed PrEP. On-demand contraceptive options have been found to be safe, acceptable, and feasible for use by women in resource-limited settings (28), and studies suggest demand for such products could be substantial (29).

Multiple preference studies with young women, their partners, and authority figures such as matriarchs suggest that the vast majority who wish to avoid pregnancy, HIV, and STIs would prefer MPTs over single-indication prevention products (30–33). Using the discrete choice experiment (DCE) method, Minnis et al. analyzed the preferences of over 500 young women in jurisdictions of Kenya and South Africa with high HIV prevalence and found that 92% would prefer an MPT for PrEP and contraception over a PrEP-only product (30). Friedland et al. found that 82% of women responding to an online survey from multiple countries, over half of whom were from sub-Saharan Africa, expressed preference for an MPT over a PrEP-only product (33). Wagner et al. found that male partners, too, tended to prefer MPTs, with a particular preference for injection over rings or oral tablets for privacy and convenience (34). Among adolescent girls and young women in South Africa, forecasts of future HIV PrEP uptake (oral, vaginal ring, injectable) increased 4-fold if products also provided pregnancy protection (35).

MPTs may offer the additional benefit of circumventing PrEP stigma, a major barrier to effective PrEP use (36–38). Many societies attach less stigma to contraception use than to PrEP use (27), allowing the contraceptive function of MPTs to serve as a proverbial “fig leaf” to divert attention away from PrEP stigma (39). A “fig leaf” could help to alleviate multiple challenges that have impeded PrEP scale-up, including internalized stigma, disapproval from partners or authority figures, the need to conceal PrEP, and fear of gender-based violence (40–42). It could also offer new opportunities to market PrEP-containing products in a broader manner than just PrEP, e.g., as a general wellness product (27, 43).

Despite these potential benefits, there remain several important challenges and barriers for effective MPTs in women not wanting to get pregnant. Contraception remains highly stigmatized in many settings, especially among adolescents and young women (44), which could erase or even reverse the “fig leaf” effect. In addition, for some, HIV prevention needs may not fully align with periods of risk for unintended pregnancy, producing unnecessary costs and side effects when only one form of prevention is needed, and potentially increasing burden on health providers due to greater need for product switching.

Product switching could also have deleterious effects on MPT cost-effectiveness, i.e., quantity of benefit per expenditure of resources when compared to other potential uses of these resources. Prior modeling studies have found that oral PrEP cost-effectiveness among most women in sub-Saharan Africa is reliant on aligning PrEP use with time periods of heightened risk (45–48). For longer-acting MPTs such as implants, changes in prevention needs or intolerable side effects from any one component of an MPT may require premature removal. For shorter-acting products, some MPTs could offer less flexibility to optimally time product use, e.g., focusing use during periods of condomless sex with a partner potentially able to transmit HIV and/or an STI, due to mismatches in timing of when PrEP and contraception can be paused and resumed while remaining safe and effective.

As no MPTs are currently licensed and many are early in the product development pipeline, MPTs are likely to cover a narrower range of product formats than single-indication products for the next several years. The daily oral Dual Prevention Pill (DPP), which co-formulates oral tenofovir/emtricitabine HIV PrEP with oral estrogen/progestin contraception, is likely to be the first MPT to be licensed. While its introduction will represent a tremendous milestone in MPTs, it will be just one initial step toward fulfilling the need for MPTs, considering that long-acting contraceptive methods are the fastest-growing segment of method mix in sub-Saharan Africa (49). Long-acting contraceptives (50) and long-acting injectable PrEP (51) have been observed to be more effective than short-acting alternatives. Long-acting products may also facilitate more effective use. For example, in a clinical crossover study of vaginally inserted PrEP products, women tried placebo versions of four products for 1 month each, and adherence was such that the long-acting monthly ring offered significantly greater PrEP coverage over time than any short-acting vaginal product (vaginal film, tablet insert, or gel) (52). Initially introducing only short-acting MPT formats could force women to choose between MPTs that are less effective for prevention, versus separate products that are, individually, more effective for prevention. A modeling study based on a DCE in South Africa suggests that adding pregnancy prevention to HIV PrEP—and, to a lesser extent, adding STI prevention—would be strongly preferred and increase PrEP use much more among adolescent girls, compared to increasing the efficacy of PrEP (35).

While women who want to prevent unintended pregnancy have been the focus of a majority of MPTs under development, gaps remain, and additional innovations are under consideration: for example, an oral MPT that does not use estrogen, thereby avoiding cardiovascular and other contraindications (53). Parallel innovations in HIV PrEP, such as a 6-monthly subcutaneous injectable method currently in development (54), could enable safer, more potent, and longer-lasting MPT options with a more preferred drug delivery format. Innovations in contraceptive administration methods, such as contraceptive self-injection using the Sayana Press device (55), could offer greater agency and lesser dependence on under-resourced health systems. These and other innovations could lead to a robust and inclusive array of MPT options for women at life stages when they wish to avoid pregnancy.

## Desiring pregnancy

MPTs that include contraception—the majority of those under development—clearly would not be indicated for women desiring pregnancy, as they would prevent conception. However, women desiring pregnancy still face barriers to PrEP and STI prevention, and could benefit from the “fig leaf” effect of MPTs—the more so because the pre-conception, conception, and pregnancy periods carry biologically elevated HIV risk (56–58) and because women desiring pregnancy would not be able to rely on condoms for HIV/STI prevention. For women who relied on MPTs to circumvent PrEP stigma, the “fig leaf” will be snatched away for each successive pregnancy. For those at sustained risk of HIV, MPTs that can only be used during life stages when a woman wishes to avoid pregnancy would result in gaps in MPT eligibility over the lifecourse, potentially postponing rather than preventing HIV infection.

For continuity of MPT benefits into the pre-conception period, product development would need to span a broader set of reproductive and health-related dimensions. Long-acting implants could emphasize switchable MPT product concepts, such as devices that could pause contraception while women desire or experience pregnancy (59, 60), thereby reducing removals and re-implantations. One long-acting reversible contraceptive implant has been designed to use an wireless controller to switch contraception on and off through the skin (61), though product developers have yet to incorporate an HIV or STI prevention component into such device concepts.

While most research on MPTs for women has focused on combined HIV prevention and contraception options, research suggests that women also place a high value on products that provide simultaneous protection from HIV and other STIs (62). Several products are currently under development, including three vaginal rings (63–65), one vaginal gel (66), and several product formats for rectal application (67–70). A modeling analysis estimating uptake of various MPTs and HIV prevention products based on DCE data from South Africa found that uptake of HIV prevention among women increased by an additional 30% if products also provided STI prevention (35). The combination of HIV and STI protection may be particularly appealing for women desiring pregnancy to avoid the risk of infertility associated with untreated STIs, as previous research has found that STIs are a leading cause of infertility in Africa (71, 72). Further research is needed to explore the preferences and motivations of this particular sub-group to inform development and prioritization of MPTs to meet their health needs.

## Pregnancy

Pregnant women bring unique opportunities and challenges for MPT development. Pregnancy is associated with heightened HIV risk (73), and maternal HIV and STI infections can cause risks to the fetus, making this time period an important opportunity to avert SRH-related health burdens across generations. Pregnancy is also time when most women have reliable contact with the healthcare system, and for some may be a first opportunity for HIV and STI screening and access to prevention services. For women testing HIV-negative in antenatal care, initiation of a life-stage-appropriate MPT could serve as a gateway to future MPT use. On the other hand, pregnancy is a time when some MPTs, including the DPP, would be contraindicated. Pregnancy also creates numerous new demands on women, including symptoms such as nausea and fatigue, increased nutritional needs, medical visits, and planning for labor, delivery, and caregiving. MPTs could allow women to integrate HIV and STI prevention into activities for other prevention needs so that HIV and STI prevention does not add further burden during this demanding life stage.

One potential MPT product concept for pregnant women could combine HIV and STI prophylaxis with a prenatal vitamin and mineral supplement, which is widely recommended from pre-conception through pregnancy and lactation. Such product carry relatively little stigma and could provide a “fig leaf” to circumvent PrEP stigma, while also avoiding adding to product burden given that prenatal supplements are universally recommended.

## Postpartum period and breastfeeding

As with pregnancy, the postpartum period is associated with heightened HIV risk (73). The postpartum and breastfeeding period is also extremely demanding on women's time and resources, and is a time when some MPTs under development, including the DPP, would be contraindicated.

It is recommended that women continue to take prenatal micronutrient supplements over the postpartum and breastfeeding period to support recovery and lactation. Thus, a micronutrient MPT could be suitable for this life stage.

In addition, postpartum and breastfeeding women may wish to reduce their risk of becoming pregnant again, either to accomplish spacing between pregnancies, or because their final family size has been achieved. Some, but not all, contraceptive-containing MPTs may be appropriate for such women. While two versions of the DPP are currently under development, both formulations contain combined hormonal contraception with estrogen, which is contraindicated for women during the first weeks after birth. MPTs that combine PrEP with contraception options that can be used immediately after birth (implants, injections, progestogen-only pills) could help meet the needs of postpartum women who are looking to delay or avoid subsequent pregnancies.

## Menopause

Peri-menopause and menopause are associated with a range of health risks in women, including cardiovascular disease, metabolic syndrome, musculoskeletal disorders, cognitive decline, depression, vasomotor symptoms, sleep disturbances, and migraine (74). Moreover, globally, an estimated 110,000 new HIV infections occurred in women aged 50 years and over, demonstrating an ongoing need for products that prevent HIV and other STIs (75).

Menopause is often associated with vaginal dryness. MPTs that combine lubrication with prevention of HIV and/or STIs could be a beneficial prevention method in this age group.

Additionally, some research suggests that estrogen therapy decreases coronary heart disease and all-cause mortality for health women aged 50–59 years (76). As a result, for women in menopause who remain at risk for HIV and other STIs, MPTs that combine estrogen therapy with STI and/or HIV prevention may provide an option for dual protection. However, treatments and health risks associated with menopause and hormone replacement remain critically understudied globally and warrant further research.

## Discussion

While MPTs offer promising opportunities to meet the health needs and preferences of women not desiring pregnancy, development of MPTs directed toward other stages of the reproductive lifecourse remains limited. We have identified a number of potentially novel product concepts (Table 1), which illustrate the opportunities for offering women continuity of MPT benefits across the reproductive lifecourse. Ultimately, product concepts should be co-created with patients, providers, and other stakeholders using a framework combining innovation, impact, and equity goals. Implementation frameworks such as the Health Equity Implementation Framework (19) and the CFIR Innovation Outcomes Addendum (20) can help guide the synthesis of patient, provider, and decision-maker factors in the context of their healthcare, sociopolitical, societal, and economic contexts toward equitable population impact. Using these frameworks, and building on the momentum of recent MPT innovations, developers and funders should evaluate MPT options that more effectively span a woman's reproductive life, particularly in vulnerable and underserved life stages such as pre-conception, pregnancy, lactation, and menopause.

**Table 1:** Challenges and opportunities for MPT product concepts across the reproductive lifespan.

Reproductive life stage	Challenges with MPT pipeline	Potential new product concepts
Not desiring pregnancy	• Unexpected prevention needs • Intermittent prevention needs • Contraindications • Side effects	• Emergency contraception + PEP • On-demand contraception + on-demand PrEP • Non-estrogen and non-hormonal
Desiring pregnancy	• Most MPTs in development include contraception	• Long-lasting switchable implants • Prenatal supplements + PrEP
Pregnancy	• Some MPTs contraindicated • Demanding life stage	• Long-lasting switchable implants • Prenatal supplements + PrEP
Postpartum/breastfeeding	• Some MPTs contraindicated • Demanding life stage • Shifting reproductive intentions, e.g., wishing to delay next pregnancy	• Long-lasting switchable implants • Prenatal supplements + PrEP • Non-estrogen and non-hormonal (if wishing to delay next pregnancy)
Menopause	• Shifting health needs and priorities	• Lubricant-based MPTs • Hormone replacement + PrEP

Beyond development and licensure, many steps remain to realize the benefits of MPTs. Once licensed, MPTs will necessitate co-delivery of multiple SRH services, which in low-resource settings often operate under separate funding sources, vertically-designed infrastructure, and siloed administrative entities (77–79). The World Health Organization (WHO) recently issued conditional recommendations to integrate HIV and family planning services (80)—an important step toward implementation—but a catalyst such as MPT introduction could accelerate action, analogous to how COVID-19 lockdowns accelerated the implementation of HIV treatment multi-month dispensation guidelines (81). Done right, MPT implementation could increase health system efficiencies by consolidating clinical visits and pharmacy dispensations.

Despite their tremendous promise, MPT introduction is likely to force difficult trade-offs in resource-limited healthcare settings. Financially, if MPTs were less cost-effective than currently available options, there is a risk that they could divert funds from other, more cost-effective health services, leading to a net detriment to population health. Similarly, given severe constraints on the number of healthcare providers in low-resource settings, if MPTs were to divert limited provider time away from other activities, potential harms would need to be weighed against potential benefits at the systems level. Interviews with Kenyan and South African healthcare providers have highlighted how MPT introduction could increase provider workload, e.g., by complicating counseling or requiring more frequent product switching (82). Providers have also raised concerns about the readiness of inventory controls to accommodate MPTs (82). Given persistent challenges with product stock-outs in low-resource settings, it is vital that MPTs not displace other product options in manners that reduce access or detriment health overall.

Licensure of the DPP—the first MPT since the condom—is likely to spark new ideas among innovators globally, including MPT users themselves. Human-centered design, co-creation, and the composition of R&D leadership should tap into the motivation and lived experiences of those most in need of MPTs. Sub-Saharan Africa should become a hub for women-led MPT innovation, as it is home to 15% of the world's women of reproductive age, 24% of women with unmet need for contraception (83), and 93% of the world's women living with HIV (84).

Although challenges and opportunities remain, women and their partners, care providers, and community leaders have expressed strong enthusiasm for MPTs already in the development pipeline. The potential benefits of these products could work across multiple levels—greater motivation at the user level, fewer product administration events at the user or provider level, accelerated delivery integration at the health systems level, and opportunities to circumvent stigma at the societal level—which could synergize to support greater access, effective use, and improved health and quality of life. The opportunity to tackle two of the leading causes of death among women of reproductive age, while honoring women's preferences and supporting intergenerational health and equity, makes MPTs one of the most promising global health frontiers of our time.

## References

[1] Vos T Lim SS Abbafati C Abbas KM Abbasi M Abbasifard M Global burden of 369 diseases and injuries in 204 countries and territories, 1990–2019: a systematic analysis for the global burden of disease study 2019. Lancet. (2020) 396(10258):1204–22. 10.1016/S0140-6736(20)30925-9 33069326 PMC7567026

[2] Karim SA Baxter C Frohlich J Karim QA. The need for multipurpose prevention technologies in sub-saharan Africa. BJOG. (2014) 121(Suppl 5):27–34. 10.1111/1471-0528.12842 25335838 PMC4206830

[3] Farrell B French Merkley V Ingar N. Reducing pill burden and helping with medication awareness to improve adherence. Can Pharm J. (2013) 146(5):262–9. 10.1177/1715163513500208 PMC3785195 24093037

[4] Nachega JB Parienti JJ Uthman OA Gross R Dowdy DW Sax PE Lower pill burden and once-daily antiretroviral treatment regimens for HIV infection: a meta-analysis of randomized controlled trials. Clin Infect Dis. (2014) 58(9):1297–307. 10.1093/cid/ciu046 24457345 PMC3982838

[5] Parati G Kjeldsen S Coca A Cushman WC Wang J. Adherence to single-pill versus free-equivalent combination therapy in hypertension: a systematic review and meta-analysis. Hypertension. (2021) 77(2):692–705. 10.1161/HYPERTENSIONAHA.120.15781 33390044

[6] Schiff M Saunderson S Mountian I Hartley P. Chronic disease and self-injection: ethnographic investigations into the patient experience during treatment. Rheumatol Ther. (2017) 4(2):445–63. 10.1007/s40744-017-0080-4 28956300 PMC5696292

[7] Meyers K Rodriguez K Brill AL Wu Y La Mar M Dunbar D Lessons for patient education around long-acting injectable PrEP: findings from a mixed-method study of phase II trial participants. AIDS Behav. (2018) 22(4):1209–16. 10.1007/s10461-017-1871-x 28744666 PMC5785575

[8] Chounta V Overton ET Mills A Swindells S Benn PD Vanveggel S Patient-reported outcomes through 1 year of an HIV-1 clinical trial evaluating long-acting cabotegravir and rilpivirine administered every 4 or 8 weeks (ATLAS-2M). Patient. (2021) 14(6):849–62. 10.1007/s40271-021-00524-0 34056699 PMC8563641

[9] Fifer S Rose J Hamrosi KK Swain D. Valuing injection frequency and other attributes of type 2 diabetes treatments in Australia: a discrete choice experiment. BMC Health Serv Res. (2018) 18(1):675. 10.1186/s12913-018-3484-0 30165844 PMC6117901

[10] Shikiar R Rentz AM. Satisfaction with medication: an overview of conceptual, methodologic, and regulatory issues. Value Health. (2004) 7(2):204–15. 10.1111/j.1524-4733.2004.72252.x 15164810

[11] Lince-Deroche N Hendrickson C Moolla A Kgowedi S Mulongo M. Provider perspectives on contraceptive service delivery: findings from a qualitative study in Johannesburg, South Africa. BMC Health Serv Res. (2020) 20(1):128. 10.1186/s12913-020-4900-9 32085756 PMC7035764

[12] Bradley SEK Polis CB Bankole A Croft T. Global contraceptive failure rates: who is most at risk? Stud Fam Plann. (2019) 50(1):3–24. 10.1111/sifp.12085 30791104 PMC6594038

[13] Izudi J Okello G Semakula D Bajunirwe F. Low condom use at the last sexual intercourse among university students in sub-Saharan Africa: evidence from a systematic review and meta-analysis. PLoS One. (2022) 17(8):e0272692. 10.1371/journal.pone.0272692 35947583 PMC9365151

[14] Nyoni P James N. Condom use and risk factors of inconsistent or low use of the condoms during heterosexual anal intercourse in sub-Saharan Africa: a scoping review. Afr Health Sci. (2022) 22(1):11–20. 10.4314/ahs.v22i1.3 36032479 PMC9382482

[15] Mazibuko NE Saruchera M Okonji EF. A qualitative exploration of factors influencing non-use of sexual reproductive health services among university students in South Africa. Int J Environ Res Public Health. (2023) 20(3):2418. 10.3390/ijerph20032418 36767788 PMC9916358

[16] Duby Z Bergh K Jonas K Reddy T Bunce B Fowler C ‘Men rule… this is the normal thing. We normalise it and it’s wrong’: gendered power in decision-making around sex and condom use in heterosexual relationships amongst adolescents and young people in South Africa. AIDS Behav. (2023) 27(6):2015–29. 10.1007/s10461-022-03935-8 36441410 PMC10149448

[17] van der Straten A Agot K Ahmed K Weinrib R Browne EN Manenzhe K The tablets, ring, injections as options (TRIO) study: what young African women chose and used for future HIV and pregnancy prevention. J Int AIDS Soc. (2018) 21(3):e25094. 10.1002/jia2.25094 29600595 PMC5876496

[18] MPT product development database. Available at: https://mpts101.org/ (Accessed February 18, 2023).

[19] Woodward EN Matthieu MM Uchendu US Rogal S Kirchner JE. The health equity implementation framework: proposal and preliminary study of hepatitis C virus treatment. Implement Sci. (2019) 14(1):26. 10.1186/s13012-019-0861-y 30866982 PMC6417278

[20] Damschroder LJ Reardon CM Opra Widerquist MA Lowery J. Conceptualizing outcomes for use with the consolidated framework for implementation research (CFIR): the CFIR outcomes addendum. Implement Sci. (2022) 17(1):7. 10.1186/s13012-021-01181-5 35065675 PMC8783408

[21] Camlin CS Koss CA Getahun M Owino L Itiakorit H Akatukwasa C Understanding demand for PrEP and early experiences of PrEP use among young adults in rural Kenya and Uganda: a qualitative study. AIDS Behav. (2020) 24(7):2149–62. 10.1007/s10461-020-02780-x 31955361 PMC7909847

[22] Yolande C Shawn M Ram P Paul N-C Jeff M Anabel G Understanding HIV prevention in high-risk adolescent girls and young women in two South African provinces. S Afr Health Rev. (2019) 2019(1):167–71.

[23] Nel A van Niekerk N Kapiga S Bekker LG Gama C Gill K Safety and efficacy of a dapivirine vaginal ring for HIV prevention in women. N Engl J Med. (2016) 375(22):2133–43. 10.1056/NEJMoa1602046 27959766

[24] Brown ER Hendrix CW van der Straten A Kiweewa FM Mgodi NM Palanee-Philips T Greater dapivirine release from the dapivirine vaginal ring is correlated with lower risk of HIV-1 acquisition: a secondary analysis from a randomized, placebo-controlled trial. J Int AIDS Soc. (2020) 23(11):e25634. 10.1002/jia2.25634 33206462 PMC7673220

[25] Miller L Prieto Merino D Baisley K Hayes R. Hidden heterogeneity: uncovering patterns of adherence in microbicide trials for HIV prevention. PLoS One. (2022) 17(5):e0267011. 10.1371/journal.pone.0267011 35551324 PMC9098085

[26] Joseph Davey D Nyemba DC Castillo-Mancilla J Wiesner L Norman J Mvududu R Adherence challenges with daily oral pre-exposure prophylaxis during pregnancy and the postpartum period in South African women: a cohort study. J Int AIDS Soc. (2022) 25(12):e26044. 10.1002/jia2.26044 36480171 PMC9731362

[27] Friedland BA Mathur S Haddad LB. The promise of the dual prevention pill: a framework for development and Introduction. Front Reprod Health. (2021) 3. 10.3389/frph.2021.682689 34318291 PMC8312733

[28] Festin MPR Bahamondes L Nguyen TMH Habib N Thamkhantho M Singh K A prospective, open-label, single arm, multicentre study to evaluate efficacy, safety and acceptability of pericoital oral contraception using levonorgestrel 1.5 mg. Hum Reprod. (2016) 31(3):530–40. 10.1093/humrep/dev341 26830816 PMC4755445

[29] Raymond EG Shochet T Drake JK Westley E. What some women want? On-demand oral contraception. Contraception. (2014) 90(2):105–10. 10.1016/j.contraception.2014.04.008 24835831

[30] Minnis AM Browne EN Boeri M Agot K van der Straten A Ahmed K Young women’s stated preferences for biomedical HIV prevention: results of a discrete choice experiment in Kenya and South Africa. J Acquir Immune Defic Syndr. (2019) 80(4):394–403. 10.1097/QAI.0000000000001945 30633040 PMC6410963

[31] Terris-Prestholt F Hanson K MacPhail C Vickerman P Rees H Watts C. How much demand for new HIV prevention technologies can we really expect? Results from a discrete choice experiment in South Africa. PLoS One. (2013) 8(12). 10.1371/journal.pone.0083193 24386160 PMC3875434

[32] Browne EN Montgomery ET Mansfield C Boeri M Mange B Beksinska M Efficacy is not everything: eliciting women’s preferences for a vaginal HIV prevention product using a discrete-choice experiment. AIDS Behav. (2020) 24(5):1443–51. 10.1007/s10461-019-02715-1 31696371 PMC6990865

[33] Friedland BA Plagianos M Savel C Kallianes V Martinez C Begg L Women want choices: opinions from the share.learn.shape global internet survey about multipurpose prevention technology (MPT) products in development. AIDS Behav. (2023). 10.1007/s10461-022-03951-8 PMC10224828 36881183

[34] Wagner LD Minnis AM Shea J Agot K Ahmed K van der Straten A. Female and male partner perspectives on placebo multipurpose prevention technologies (MPTs) used by women in the TRIO study in South Africa and Kenya. PLoS One. (2022) 17(5):e0265303. 10.1371/journal.pone.0265303 35551318 PMC9097999

[35] Vickerman P Quaife M Kilbourne-Brook M Mvundura M Eakle R Terris-Prestholt F. HIV prevention is not all about HIV - using a discrete choice experiment among women to model how the uptake and effectiveness of HIV prevention products may also rely on pregnancy and STI protection. BMC Infect Dis. (2020) 20(1). 10.1186/s12879-020-05399-4 32977745 PMC7517801

[36] Velloza J Khoza N Scorgie F Chitukuta M Mutero P Mutiti K The influence of HIV-related stigma on PrEP disclosure and adherence among adolescent girls and young women in HPTN 082: a qualitative study. 2020; Available at: https://urldefense.com/v3/__http://onlinelibrary.wiley.com/doi/10.1002/jia2.25463/full__;!!MXfaZl3l!ZF1GetJ-uHTqLdzjJ9IqgN09yQHEM2R85-lhyxSAuU_ACX4llk2wBf0w_blbHn_dkuXXBIgUF0kicPfnpkIZ2VARB4zvgHUU$. 10.1002/jia2.25463 PMC7060297 32144874

[37] Karuga RN Njenga SN Mulwa R Kilonzo N Bahati P O’Reilley K ‘How i wish this thing was initiated 100 years ago!’ willingness to take daily oral pre-exposure prophylaxis among men who have sex with men in Kenya. PLoS One. (2016) 11(4). 10.1371/journal.pone.0151716 27073896 PMC4830617

[38] Magazi B Stadler J Delany-Moretlwe S Montgomery E Mathebula F Hartmann M Influences on visit retention in clinical trials: insights from qualitative research during the VOICE trial in Johannesburg, South Africa. BMC Womens Health. (2014) 14(1). 10.1186/1472-6874-14-88 25065834 PMC4115485

[39] Tsui AO Brown W Li Q. Contraceptive practice in sub-Saharan Africa. Popul Dev Rev. (2017) 43:166–91. 10.1111/padr.12051 29081552 PMC5658050

[40] Patel RC Leddy AM Odoyo J Anand K Stanford-Moore G Wakhungu I What motivates serodiscordant couples to prevent HIV transmission within their relationships: findings from a PrEP implementation study in Kenya. Cult Health Sex. (2018) 20(6):625–39. 10.1080/13691058.2017.1367421 28903628 PMC5851810

[41] Barnabee G O’Bryan G Ndeikemona L Billah I Silas L Morgan KL Improving HIV pre-exposure prophylaxis persistence among adolescent girls and young women: insights from a mixed-methods evaluation of community, hybrid, and facility service delivery models in Namibia. Front Reprod Health. (2022) 4:1048702. 10.3389/frph.2022.1048702 36545490 PMC9760915

[42] Katz AWK Roberts S Rousseau E Khoza MN Mogaka F Bukusi E Qualitative analysis using social maps to explore young women’s experiences with social support of their oral PrEP use in Kenya and South Africa. J Assoc Nurses AIDS Care. (2023) 34(1):45–57. 10.1097/JNC.0000000000000363 36170124

[43] Rosen JG Toomre T To C Olatunde PF Cooper L Glick JL Communicative appeals and messaging frames in visual media for HIV pre-exposure prophylaxis promotion to cisgender and transgender women. Cult Health Sex. (2022):1–17. 10.1080/13691058.2022.2116111 36074902 PMC9992445

[44] Jonas K Duby Z Maruping K Dietrich J Slingers N Harries J Perceptions of contraception services among recipients of a combination HIV-prevention interventions for adolescent girls and young women in South Africa: a qualitative study. Reprod Health. (2020) 17(1):122. 10.1186/s12978-020-00970-3 32795366 PMC7427945

[45] Pretorius C Schnure M Dent J Glaubius R Mahiane G Hamilton M Modelling impact and cost-effectiveness of oral pre-exposure prophylaxis in 13 low-resource countries. J Int AIDS Soc. (2020) 23(2):e25451. 10.1002/jia2.25451 32112512 PMC7048876

[46] Vogelzang M Terris-Prestholt F Vickerman P Delany-Moretlwe S Travill D Quaife M. Cost-effectiveness of HIV Pre-exposure prophylaxis among heterosexual men in South Africa: a cost-utility modeling analysis. J Acquir Immune Defic Syndr. (2020) 84(2):173–81. 10.1097/QAI.0000000000002327 32141959

[47] Roberts DA Bridenbecker D Haberer JE Barnabas RV Akullian A. The impact of prevention-effective PrEP use on HIV incidence: a mathematical modelling study. J Int AIDS Soc. (2022) 25(11):e26034. 10.1002/jia2.26034 36385504 PMC9670193

[48] Phillips AN Bershteyn A Revill P Bansi-Matharu L Kripke K Boily MC Cost-effectiveness of easy-access, risk-informed oral pre-exposure prophylaxis in HIV epidemics in sub-saharan Africa: a modelling study. Lancet HIV. (2022) 9(5):e353–62. 10.1016/S2352-3018(22)00029-7 35489378 PMC9065367

[49] Haakenstad A Angelino O Irvine CMS Bhutta ZA Bienhoff K Bintz C Measuring contraceptive method mix, prevalence, and demand satisfied by age and marital status in 204 countries and territories, 1970–2019: a systematic analysis for the global burden of disease study 2019. Lancet. (2022) 400(10348):295–327. 10.1016/S0140-6736(22)00936-9 35871816 PMC9304984

[50] Festin MPR. Overview of modern contraception. Best Pract Res Clin Obstet Gynaecol. (2020) 66:4–14. 10.1016/j.bpobgyn.2020.03.004 32291177

[51] Delany-Moretlwe S Hughes JP Bock P Ouma SG Hunidzarira P Kalonji D Cabotegravir for the prevention of HIV-1 in women: results from HPTN 084, a phase 3, randomised clinical trial. Lancet. (2022) 399(10337):1779–89. 10.1016/S0140-6736(22)00538-4 35378077 PMC9077443

[52] Montgomery ET Beksinska M Mgodi N Schwartz J Weinrib R Browne EN End-user preference for and choice of four vaginally delivered HIV prevention methods among young women in South Africa and Zimbabwe: the quatro clinical crossover study. J Int AIDS Soc. (2019) 22(5):e25283. 10.1002/jia2.25283 31069957 PMC6506690

[53] Zuniga C Blanchard K Harper CC Wollum A Key K Henderson JT. Effectiveness and efficacy rates of progestin-only pills: a comprehensive literature review. Contraception. (2023) 119:109925. 10.1016/j.contraception.2022.109925 36535414

[54] Overmars RJ Krullaars Z Mesplède T. Investigational drugs for HIV: trends, opportunities and key players. Expert Opin Investig Drugs. (2023) 32:2. 10.1080/13543784.2023.2178415 36751107

[55] Burke HM Chen M Packer C Fuchs R Ngwira B. Young women’s experiences with subcutaneous depot medroxyprogesterone acetate: a secondary analysis of a one-year randomized trial in Malawi. J Adolesc Health. (2020) 67(5):700–7. 10.1016/j.jadohealth.2020.03.038 32389457

[56] Drake AL Wagner A Richardson B John-Stewart G. Incident HIV during pregnancy and postpartum and risk of mother-to-child HIV transmission: a systematic review and meta-analysis. PLoS Med. (2014) 11(2):e1001608. 10.1371/journal.pmed.1001608 24586123 PMC3934828

[57] Machekano R Tiam A Kassaye S Tukei V Gill M Mohai F HIV incidence among pregnant and postpartum women in a high prevalence setting. PLoS One. (2018) 13(12):e0209782. 10.1371/journal.pone.0209782 30592749 PMC6310250

[58] Mussa A Mayondi GK Diseko M Mabuta J Mmalane M Makhema J Incident HIV acquisition among pregnant women in Botswana: findings from the Tsepamo birth outcomes surveillance study. J Int AIDS Soc. (2023) 26(1):e26008. 10.1002/jia2.26008 36691796 PMC9871722

[59] A contraceptive implant with remote control. MIT technology review. Available at: https://www.technologyreview.com/2014/07/04/74389/a-contraceptive-implant-with-remote-control/ (Accessed February 17, 2023).

[60] Sadeghi I Byrne J Shakur R Langer R. Engineered drug delivery devices to address global health challenges. J Control Release. (2021) 331:503–14. 10.1016/j.jconrel.2021.01.035 33516755 PMC7842133

[61] Subcutaneous insertion and removal of a user-controlled, long-acting, and reversible contraceptive device. SBIR.gov. Available at: https://www.sbir.gov/node/2192021 (Accessed April 17, 2023).

[62] Hynes JS Sales JM Sheth AN Lathrop E Haddad LB. Interest in multipurpose prevention technologies to prevent HIV/STIs and unintended pregnancy among young women in the United States. Contraception. (2018) 97(3):277–84. 10.1016/j.contraception.2017.10.006 29055782 PMC5828781

[63] Thurman AR Ravel J Gajer P Marzinke MA Ouattara LA Jacot T Vaginal microbiota and mucosal pharmacokinetics of tenofovir in healthy women using a 90-day tenofovir/levonorgestrel vaginal ring. Front Cell Infect Microbiol. (2022) 12. 10.3389/fcimb.2022.799501 35350436 PMC8957918

[64] Janusziewicz R Mecham SJ Olson KR Benhabbour SR. Design and characterization of a novel series of geometrically complex intravaginal rings with digital light synthesis. Adv Mater Technol. (2020) 5(8):2000261. 10.1002/admt.202000261 33072856 PMC7567335

[65] Smith JM Moss JA Srinivasan P Butkyavichene I Gunawardana M Fanter R Novel multipurpose pod-intravaginal ring for the prevention of HIV, HSV, and unintended pregnancy: pharmacokinetic evaluation in a macaque model. PLoS One. (2017) 12(10):e0185946. 10.1371/journal.pone.0185946 28982161 PMC5628903

[66] North BB Weitzel MB Waller DP Birch WX Feathergill KA Birch LA Evaluation of the novel vaginal contraceptive agent PPCM in preclinical studies using sperm hyaluronan binding and acrosome status assays. Andrology. (2022) 10(2):367–76. 10.1111/andr.13110 34542939 PMC8760152

[67] Hiruy H Fuchs EJ Marzinke MA Bakshi RP Breakey JC Aung WS A phase 1 randomized, blinded comparison of the pharmacokinetics and colonic distribution of three candidate rectal microbicide formulations of tenofovir 1% gel with simulated unprotected sex (CHARM-02). AIDS Res Hum Retroviruses. (2015) 31(11):1098–108. 10.1089/aid.2015.0098 26227279 PMC4651050

[68] Ho K Hoesley C Anderson P Kelly C Jiao Y Edick S Phase 1 safety and pharmacokinetic study of candidate rectal microbicide PC-1005 rectal gel (MIV-150/zinc acetate/carrageenan) in HIV-1 seronegative adults (MTN-037). J Int AIDS Soc. (2021) 24(S1):40–2. 10.1002/jia2.25659

[69] Gao Z Fu R Li X Wang J He Y. Safety assessment of microbicide 2P23 on the rectal and vaginal microbiota and its antiviral activity on HIV infection. Front Immunol. (2021) 12:374–92. 10.3389/fimmu.2021.702172 PMC8382973 34447373

[70] Makarova N Singletary T Peet MM Mitchell J Holder A Dinh C Pharmacokinetics and efficacy of topical inserts containing tenofovir alafenamide fumarate and elvitegravir administered rectally in macaques. eBioMedicine. (2022) 86:104338. 10.1016/j.ebiom.2022.104338 36343572 PMC9643401

[71] Larsen U. Infertility in central Africa. Trop Med Int Health. (2003) 8(4):354–67. 10.1046/j.1365-3156.2003.01039.x 12667156

[72] Gottlieb SL Low N Newman LM Bolan G Kamb M Broutet N. Toward global prevention of sexually transmitted infections (STIs): the need for STI vaccines. Vaccine. (2014) 32(14):1527–35. 10.1016/j.vaccine.2013.07.087 24581979 PMC6794147

[73] Thomson KA Hughes J Baeten JM John-Stewart G Celum C Cohen CR Increased risk of HIV acquisition among women throughout pregnancy and during the postpartum period: a prospective per-coital-act analysis among women with HIV-infected partners. J Infect Dis. (2018) 218(1):16–25. 10.1093/infdis/jiy113 29514254 PMC5989601

[74] van Dijk GM Kavousi M Troup J Franco OH. Health issues for menopausal women: the top 11 conditions have common solutions. Maturitas. (2015) 80(1):24–30. 10.1016/j.maturitas.2014.09.013 25449663

[75] AIDSinfo. UNAIDS. Available at: https://aidsinfo.unaids.org/ (Accessed April 17, 2023).

[76] Lobo RA Davis SR De Villiers TJ Gompel A Henderson VW Hodis HN Prevention of diseases after menopause. Climacteric. (2014) 17(5):540–56. 10.3109/13697137.2014.933411 24969415

[77] Mayhew SH Hopkins J Warren CE. Building integrated health systems: lessons from HIV, sexual and reproductive health integration. Health Policy Plan. (2017) 32(suppl_4):iv1–5. 10.1093/heapol/czx142 29194546 PMC5886152

[78] Nkhoma L Sitali DC Zulu JM. Integration of family planning into HIV services: a systematic review. Ann Med. (2022) 54(1):393–403. 10.1080/07853890.2021.2020893 35098814 PMC8812772

[79] Wilcher R Cates W Gregson S. Family planning and HIV: strange bedfellows no longer. AIDS. (2009) 23(Suppl 1):S1–6. 10.1097/01.aids.0000363772.45635.35 20081381 PMC3516801

[80] World Health Organization. Consolidated guidelines on HIV prevention, testing, treatment, service delivery and monitoring: recommendations for a public health approach (2021). Available at: https://www.who.int/publications-detail-redirect/9789240031593 (Accessed August 20, 2021). 34370423

[81] Bailey LE Siberry GK Agaba P Douglas M Clinkscales JR Godfrey C. The impact of COVID-19 on multi-month dispensing (MMD) policies for antiretroviral therapy (ART) and MMD uptake in 21 PEPFAR-supported countries: a multi-country analysis. J Int AIDS Soc. (2021) 24(Suppl 6):e25794. 10.1002/jia2.25794 34713578 PMC8554217

[82] Lutnick A Shapley-Quinn MK Manenzhe KN Onyango J Agot K Ahmed K Two birds with one stone: health care providers’ perspectives about prevention technologies in Kenya and South Africa. J Int Assoc Provid AIDS Care. (2019) 18:2325958219841366. 10.1177/2325958219841366 31018754 PMC6748465

[83] Bearak JM Popinchalk A Beavin C Ganatra B Moller AB Tunçalp Ö Country-specific estimates of unintended pregnancy and abortion incidence: a global comparative analysis of levels in 2015–2019. BMJ Global Health. (2022) 7(3):e007151. 10.1136/bmjgh-2021-007151 35332057 PMC8943721

[84] UNAIDS. 20.2 Million girls and women living with HIV (2022). Available at: https://www.unaids.org/en/resources/infographics/girls-and-women-living-with-HIV (Accessed June 14, 2022).

